# The addition of autologous stem cell transplantation to neoadjuvant chemotherapy, radiation, and HIPEC for patients with unresectable desmoplastic small round cell tumor: a single center case series

**DOI:** 10.1097/ij9.0000000000000095

**Published:** 2021-01-20

**Authors:** Amir Siddiqui, Navin Pinto, Mark A. Applebaum, Grace Z. Mak, John M. Cunningham, James L. LaBelle, Michele L. Nassin

**Affiliations:** a Department of Pediatrics, Section of Hematology/Oncology/Stem Cell Transplantation, University of Chicago, Chicago, IL,; b Department of Pediatrics, Division of Hematology/Oncology, University of Washington, Seattle, WA; c Department of Surgery, Section of Pediatric Surgery, University of Chicago, Chicago, IL

**Keywords:** Desmoplastic small round cell tumor, Autologous stem cell transplantation, Hyperthermic intraperitoneal chemotherapy

## Abstract

Desmoplastic small round cell tumor (DSRCT) is a rare, highly aggressive malignancy primarily affecting children and young adults. Although modest improvements have been gained by intensification of chemotherapy and radiation, survival of patients with DSRCT remains poor, particularly in those with unresectable or disseminated disease. We report 3 pediatric patients who were treated with a combination of therapy including chemotherapy, surgical debulking, hyperthermic intraperitoneal chemotherapy, whole abdominal irradiation, and autologous hematopoietic stem cell transplantation following busulfan and melphalan conditioning. We find that this approach is well tolerated and may offer improved survival in patients with DSRCT.

## Introduction

DSRCT is a rare, highly aggressive mesenchymal tumor with dismal patient outcomes. Reported median progression-free survival (PFS) and overall survival (OS) range from 4 to 21 and 14 to 60 months, respectively^[1–11]^. It is therefore imperative to develop novel approaches to treatment for this entity. DSRCT is characterized by the t (11;22)(p13;q12) translocation resulting in a fusion *EWSR1-WT1* gene product that drives tumorigenesis^[12]^. DSRCT is most commonly diagnosed in adolescent and young adult males and typically presents as an abdominal mass with peritoneal seeding and metastases^[1,13]^. Treatment has included chemotherapy, surgical resection, and radiotherapy^[1,13,14]^. Recent studies suggest that HIPEC can be safely added to this regimen and may result in improved outcomes, particularly for patients with residual disease following surgical debulking measuring <2.5 cm^[5–8]^. Independently, the addition of autoHSCT to chemotherapy, surgical resection and radiation has also been investigated and may improve overall survival in patients who achieved complete remission before transplant^[3]^. It is imperative to develop new strategies to effectively manage this disease that has historically had such dismal outcomes. Our center hypothesized that the addition of both HIPEC and autoHSCT to neoadjuvant chemotherapy, surgical resection, and radiation would be well tolerated and improve outcomes in patients with DSRCT and residual disease following surgical resection.

## Materials and methods

An IRB approved (IRB 12362A) retrospective review was performed to identify patients diagnosed with DSRCT and treated consecutively at the University of Chicago Comer Children’s Hospital, an urban academic tertiary care center, between 2013 and 2018. All patients diagnosed with metastatic DSRCT during this time period were offered treatment from their clinical team that included neoadjuvant chemotherapy, surgical resection with HIPEC, radiation, and autologous HSCT. All patients were treated with HIPEC. Surgical cytoreduction was performed with preoperative goal of complete resection when possible. This occurred in the operating room (OR), under general anesthesia in the supine position. HIPEC was performed following cytoreduction with closed technique using cisplatin at 100 mg/m^2^ (maximum dose 130 mg) for 90 minutes as previously described^[6]^. Each procedure was performed by the same experienced oncology surgeon who received specific training in the HIPEC procedure to avoid interoperator variability. Following the procedure, all patients received patient controlled analgesia with intravenous narcotics and scheduled antiemetic medications. Patients were followed closely by physical therapy and offered social work and psychological support during the treatment. All patients considered eligible for this therapy were required to be chemotherapy responsive and have good performance status upon entering the procedure so no additional pre-intervention therapy was required to optimize the patients before the OR. The use of these specific chemotherapy agents is considered off label as they are not specifically approved for DSRCT, however, they are commonly used in this patient population. Patients were monitored with surveillance CT scans to assess response following their therapy.

Patient data was abstracted from the electronic medical record. All therapy-related toxicities were graded based on the National Cancer Institute’s Common Terminology Criteria for Adverse Events version 5.0. Neutrophil and platelet engraftment were defined as the first of 3 consecutive days with an absolute neutrophil count > 500 × 10^6^/L and the first of 3 consecutive days with a platelet count of > 20 × 10^9^/L, respectively, as defined by the Center for International Blood and Marrow Transplant Research (CIBMTR). Data is reported in line with PROCESS 2018 criteria^[15]^.

## Results

### Case 1

**Figure 1.**
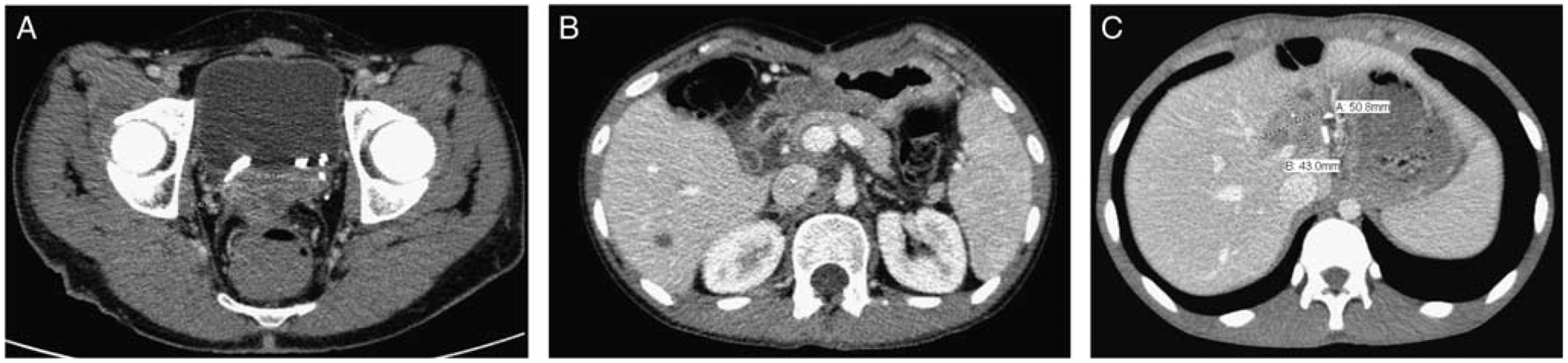
Post therapy disease imaging. Disease evaluation following completion of therapy. A, Residual recto-vesical mass noted following autoHSCT in patient 1. B, Residual hepatic lesion noted following autoHSCT in patient. C, Residual periportal lesion noted following autoHSCT in patient.

A previously healthy 17-year-old male presented with abdominal pain, weight loss, and vomiting. He was found to have multiple pleural, hepatic, pelvic, and intra-abdominal masses causing moderate compression of the portal vein and bilateral hydronephrosis requiring urgent bilateral ureteral stent placement. The largest areas of primary disease included an intra-abdominal retroperitoneal mass measuring 14.9 × 12.9 cm and a retrovesicular mass measuring 12.4 × 9.9 cm. Biopsy revealed small round blue cells and cytogenetics confirmed the *EWSR1*-*WT1* translocation. He was diagnosed with stage 4 DSRCT and received 6 cycles of vincristine, etoposide, ifosfamide, and doxorubicin (VIDE) followed by 1 cycle of vincristine, dactinomycin and ifosfamide (VAI) during surgical planning. Presurgical computed tomography (CT) scans demonstrated resolution of his pleural masses and a decrease in size of his multiple abdominal and pelvic masses. He underwent surgical debulking and omentectomy with resection of multiple hepatic, intrabdominal and pelvic masses, and removal of over 3800 nodules from the omentum, peritoneum, mesentery, and diaphragm. However, due to adherence to the portal vein, a large (4.8 × 4.3 cm) hepatic mass in the porta hepatis was debulked with removal of ~50% of tumor but not fully resected. The large pelvic mass was debulked leaving no significant gross disease over 1 cm in depth or thickness. Immediately following cytoreduction, the patient was cooled and warmed intraperitoneal cisplatin was infused. Blood loss was estimated to be ~1 L. He suffered no immediate surgical complications. The Peritoneal Cancer Index (PCI) was 26. Postsurgical CT scans demonstrated a residual porta hepatis mass measuring 1.9 by 1.4 cm and a recto-vesical mass measuring 2.8 × 4.3 cm (Fig. 1). Twelve weeks following surgery and HIPEC, the patient received 30 Gy conventional whole abdominal radiation with a 6 Gy boost to residual tumor sites. He underwent autologous stem cell transplant 19 weeks following completion of his radiotherapy. The transplant was originally planned for 6 weeks following his irradiation but was delayed by an additional 13 weeks due to dental extraction and family preference. He received conditioning chemotherapy with dose-adjusted busulfan (0.8 mg/kg q6 h for 16 doses on days − 6 to − 2 adjusted based on drug level and pharmacokinetics) and melphalan (140 mg/m^2^ on day − 2) followed by an autologous stem cell infusion of GCSF-mobilized cryopreserved stem cells [5.5 × 10^8^ total nucleated cells (TNC)/kg]. The patient had neutrophil engraftment on day + 11, platelet engraftment on day + 16, and became pRBC transfusion independent by day + 9. Treatment complications included ifosfamide encephalopathy successfully treated with methylene blue and grade 3 mucositis during transplant. Infections included *Candida* and *Enterococcus* urinary tract infections attributed to his ureteral stents and *Staphylococcus hominis* bacteremia following surgical debulking and HIPEC. All infections were successfully treated. Posttherapy complications included grade 2 chronic kidney disease, likely secondary to his tumor-induced hydronephrosis and his exposure to intraperitoneal cisplatin. His renal injury resulted in the need for long-term electrolyte repletion, but never required dialysis or additional intervention. Surveillance CT scans 23 months postdiagnosis and 9.5 months posttransplant revealed progression of his residual recto-vesical and hepatic masses and a new mass inferior to the spleen. There was no extra-abdominal disease identified at this time. Until this point, surveillance CT scans demonstrated stable residual disease without evidence of progression. He received 4 cycles of vincristine, irinotecan, and temozolomide (VIT) following the scans that identified progression. Because of continued progression of his disease despite VIT therapy, treatment was changed to the multikinase inhibitor pazopanib. Two months after initiation of pazopanib, he progressed and was transitioned to hospice care. The patient died of progressive disease at 37 months after diagnosis and 23.5 months post autoHSCT.

### Case 2

A 20-year-old male presented with abdominal pain and was found to have widespread carcinomatosis involving the thoracic, abdominal, and pelvic cavities in addition to multiple hepatic masses, and involvement of the mesenteric, retroperitoneal, and mediastinal lymph nodes. Biopsy revealed small round blue cells with a nested growth pattern in a fibrotic stroma and cytogenetics confirmed the *EWSR1*-*WT1* translocation reflecting stage 4 DSRCT. He received neoadjuvant chemotherapy with 6 cycles of VIDE. Postinduction CT scans showed resolution of his thoracic disease and a decrease in size of his abdominal and pelvic disease. He then underwent surgical resection and debulking including removal of over 7400 nodules from the omentum, peritoneum, mesentery, diaphragms, appendix, retroperitoneum, and pelvis in addition to resection of multiple hepatic masses. There were a number of unresectable small nodules due to their adherence to the bowel wall, all <5 mm in size and thickness. Immediately following cytoreduction, the patient was cooled and warmed intraperitoneal cisplatin was infused. Blood loss was estimated to be ~1 L. He suffered no immediate surgical complications. The PCI was 27. The surgery was immediately followed by HIPEC with cisplatin. Postsurgical CT scans demonstrated a residual hepatic mass measuring 0.9 × 1.5 cm (Fig. 1). Unresected nodules adherent to the bowel were not detected on postoperative CT scan. Eight weeks following surgery he underwent 30 Gy conventional whole abdomen radiation. Six weeks following completion of radiotherapy he underwent autoHSCT. The patient was conditioned with dose-adjusted busulfan (0.8 mg/kg q6 h for 16 doses on days − 6 to − 2 adjusted based on drug level and pharmacokinetics) and melphalan (140 mg/m^2^ on day − 2) followed by infusion of GCSF-mobilized cryopreserved stem cells (1.5 × 10^8^ TNC/kg). He achieved neutrophil engraftment on day + 9 poststem cell infusion, platelet engraftment on day + 14, and became pRBC transfusion independent by day + 16. Aside from posttransplant grade 3 mucositis, he had no significant therapy-related morbidity. Surveillance CT scans revealed relapsed disease with a mass anterior to the right psoas muscle, 2 prerectal lesions, and multiple lymph nodes in the chest 34.5 months postdiagnosis and 25 months posttransplant. He was then treated with 6 cycles of VIT. He transitioned to pazopanib due to significant gastrointestinal toxicity related to salvage chemotherapy. After 2 months of pazopanib his disease progressed, and he was enrolled in a clinical trial with nivolumab (IgG4 monoclonal antibody targeting PD-1), cabiralizumab (monoclonal antibody targeting CSF1R), and stereotactic body radiotherapy. He died from progressive disease 58.5 months following diagnosis and 49 months post autoHSCT.

### Case 3

A 15-year-old male presented with abdominal distension and was found to have 2 large intra-abdominal masses involving the porta hepatis and abutting the portal vein. Biopsy revealed small round blue cells within a desmoplastic stroma. Cytogenetics confirmed rearrangement of the *EWSR1* gene. He was diagnosed with stage 3 DSRCT and received six cycles of VIDE. Because of a delay related to surgical planning and patient preference, VIDE was followed by 4 cycles of VAI. Nine months following diagnosis he underwent surgical resection including removal of over 300 nodules from the omentum, peritoneum, mesentery, diaphragm, and pelvis in addition to resection of his 2 large masses. The mass involving the porta hepatis was only partially resected due to its encasing the portal vein and hepatic artery as well as involving the left gastric artery. Approximately 50% of the mass was resected. Immediately following cytoreduction, the patient was cooled and warmed intraperitoneal cisplatin was infused. Blood loss was estimated to be ~1 L. He suffered no immediate surgical complications. The PCI was 22. A postsurgical CT scan confirmed the presence of the unresectable residual porta hepatis mass measuring 6.2 × 4.4 × 4.7 cm (Fig. 1). He received 30 Gy conventional whole abdominal radiation ten weeks following surgery. Eight weeks after completion of his radiation he underwent myeloablative chemotherapy with dose-adjusted busulfan (0.8 mg/kg q6 h for 16 doses on days − 7 to − 3 adjusted based on drug level and pharmacokinetics) and melphalan (140 mg/m^2^ on day − 3) followed by infusion of GCSF-mobilized cryopreserved stem cells (8.4 × 10^8^ TNC/kg). He achieved neutrophil engraftment on day + 9 poststem cell infusion, platelet engraftment on day + 15, and became pRBC transfusion independent by day + 14. Overall complications included *Streptococcus mitis* bacteremia during neoadjuvant chemotherapy and *Lactobacillus rhamnosus* bacteremia posttransplant, both successfully treated with antibiotics. He also had grade 3 mucositis posttransplant. He had no therapy-related morbidity. The patient developed progressive intrabdominal disease 32.5 months postdiagnosis and 18.5 months posttransplant. Despite additional chemotherapy at an outside institution, he died from disease 37.5 months following diagnosis and 23.5 months post autoHSCT.

## Discussion

We report the treatment of 3 patients with advanced stage, unresectable DSRCT treated with a combination of neoadjuvant chemotherapy, surgery, HIPEC, radiation, and autoHSCT. Treatment was well tolerated and resulted in a median PFS of 32.5 months (mean 30 mo) and a median OS of 37.5 months (mean 44 mo). Previous studies have reported a range for median PFS from 4 to 21 months and median OS from 14 to 60 months using other treatments regimens some including either HIPEC or autoHSCT, although not in combination (Table 1)^[1–11,14,16]^. Despite having longer PFS, OS remained poor in our cohort.

Residual disease after cytoreductive surgery is a known poor prognostic marker. Studies that report OS > 35 months limited inclusion of patients without extra-abdominal disease at diagnosis or only included patients who achieved a complete cytoreduction after surgery^[5,7–9]^. In one study investigating HIPEC, median OS for patients without residual disease was 31.1 months while median OS was 12.8 months for those with > 2.5 cm of residual disease. Similarly, in a study investigating autoHSCT, median OS was 36 months for those without residual disease compared with 21 months for those with residual disease. Compared with patients with residual disease after surgery our patients had longer median OS at 37.5 months (Table 2).

The outcomes seen with this therapy in this cohort of patients suggests that the regimen is well tolerated and may slow progression. Therefore, we believe this regimen should be considered in patients with DSRCT given its dismal prognosis, although thorough and careful counseling with the patient is imperative.

Despite encouraging results, the small cohort size limits any conclusions or statistically significant comparisons that can be drawn about improved outcomes by combining HIPEC and autoHSCT in patients with DSRCT. Furthermore, all our patients had intrahepatic/portal disease at diagnosis and had intra-abdominal disease at relapse despite having received HIPEC, consistent with previous reports noting the limitation of HIPEC in patients with intrahepatic/portal disease^[6]^. Further prospective studies with additional patients are needed to better understand the implications of such combination therapy. As outcomes for patients with DRSCT are poor, particularly those with residual disease after surgery, novel therapeutics are needed.

## Figures and Tables

**Table 1:** Reported outcomes of patients with DSRCT.

Study	Years	N	Treatment	PFS/DFS/RFS	OS
University of Chicago Cohort	2014–2018	3	Chemotherapy, surgery, HiPEC, radiation, autoHSCT	1 y PFS 100%, 3y PFS 33%, median PFS 32.5 mo (mean 30 mo)	3y OS 100%, median OS 37.5 mo (mean 44 mo)
Subbiah et al^[16]^	1990–2016	187	Chemotherapy, + / − surgery, radiation, HiPEC, and/or autoHSCT	Not reported	3y OS 51%, 5 y OS 25%
Honoroe et al^[8]^*	1991–2015	107	Chemotherapy, surgery + / − radiation and/or HiPEC	2y DFS 30%, 5y DFS 12%, median DFS 21 mo	2y OS 72%, 5y OS 19%, median OS 42 mo
Osborne et al^[9]^*	2006–2014	32	Chemotherapy, surgery, HiPEC, whole abdominal radiation	3y PFS 10%, median PFS 10 mo	3y OS 64%, 5y OS 38%, median OS 60 mo
Zhang et al^[11]^	2004–2014	11	Chemotherapy, surgery and/or radiation	3y PFS 27%, median PFS 8.8 mo	3y OS 36.4%, 5y OS 10%, median OS 29 mo
Hayes-Jordan et al^[7]^*	2012–2013	14	Chemotherapy, surgery, HiPEC, radiation	Median RFS 14.87 mo	3y OS 79%, median OS 58.44 mo
Wong et al^[10]^	1991–2012	41	Chemotherapy, + / − surgery and/or radiation	Median PFS 4 mo	Median OS 16 mo
Hayes-Jordan et al^[6]^	2006–2011	26	Chemotherapy, surgery, HiPEC + / − radiation	1 y DFS 42% vs. 0% for those with disease limited to the abdominal cavity vs. those with disease outside the abdomen	Median OS 31.1 mo vs. 12.8 mo if patients achieved CR vs. only PR after surgical resection
Bent et al^[1]^	1991–2011	95	Chemotherapy, surgery, + / − radiation	Not reported	2y OS 52.4%, 5y OS 18.1%, median OS 25 mo
Hayes-Jordan et al^[5]^	1995–2008	24	Chemotherapy, surgery and/or radiation, + / − HiPEC	1 y PFS 53%, median PFS 12.3 mo	1 y OS 100%, 3y OS 71%,
Cooke et al^[3]^	1999–2007	36	Chemotherapy, surgery and/or radiation, autoHSCT	1 y PFS 53%, 3y PFS 23%	1 y OS 83%, 3y OS 40%, median OS 31 mo
Lal et al^[14]^	1972–2003	66	Chemotherapy, + / − surgery and/or radiation	Not reported	3y OS 44%, 5 y OS 15%
Bertuzzi et al^[2]^	1997–2002	10	Chemotherapy, surgery and/or radiation, autoHSCT	1 y PFS 12.5%	Median OS 14 mo
Goodman et al^[4]^	1992–2001	21	Chemotherapy, surgery, whole abdominal radiation, autoHSCT	3y PFS 19%, median PFS 19 mo	3y OS 48%, median OS 32 mo

* Only includes patients with a complete response after surgical resection.

AutoHSCT indicates autologous hematopoietic stem cell transplantation; CR, complete response; DFS, disease-free survival; HiPEC, hyperthermic intraperitoneal chemotherapy; OS, overall survival; PFS, progression-free survival; PR, partial response; RFS, recurrence-free survival.

**Table 2:** Patient characteristics and outcome.

Patient	Age (y)/Sex	Disease Location	EWSR1 Gene Mutation	PCI	Disease Response	PFS (mo)	OS (mo)
1	17/M	Multiple pleural, hepatic, pelvic, and intra-abdominal masses	Present	26	PR	23	37
2	20/M	Multiple hepatic masses, and involvement of the mesenteric, retroperitoneal and mediastinal lymph nodes	Present	27	PR	34.5	58.5
3	15/M	2 large intra-abdominal masses involving the porta hepatis and abutting the portal vein	Present	22	PR	32.5	37.5

M indicates male; PCI, Peritoneal Cancer Index; PFS, progression-free survival.

## Keypoints

1. Desmoplastic small round cell tumor (DSRCT) is a rare, highly aggressive malignancy with poor outcomes requiring ongoing studies to improve survival.
2. Hyperthermic intraperitoneal chemotherapy (HIPEC) and autologous hematopoietic stem cell transplantation (autoHSCT) can be safely added to the traditional therapy for DSRCT.
3. HIPEC is well tolerated in patients treated for DSRCT.
